# Retraction Note: Biosynthesis of Zn-doped CuFe_2_O_4_ nanoparticles and their cytotoxic activity

**DOI:** 10.1038/s41598-023-36215-z

**Published:** 2023-06-06

**Authors:** Maryam Darvish, Navid Nasrabadi, Farnoush Fotovat, Setareh Khosravi, Mehrdad Khatami, Samira Jamali, Elnaz Mousavi, Siavash Iravani, Abbas Rahdar

**Affiliations:** 1 Department of Endodontics, School of Dentistry, Kerman University of Medical Sciences, Kerman, Iran; 2 Department of Endodontics, School of Dentistry, Birjand University of Medical Sciences, Birjand, Iran; 3 Department of Prosthodontics, School of Dentistry, Hamadan University of Medical Sciences, Hamadan, Iran; 4 Department of Orthodontics, School of Dentistry, Alborz University of Medical Sciences, Karaj, Iran; 5 Department of Medical Biotechnology, Faculty of Medical Sciences, Tarbiat Modares University, Tehran, Iran; 6 Department of Endodontics, Stomatological Hospital, College of Stomatology, Xi’an Jiaotong University, Shaanxi, 710004 People’s Republic of China; 7 Dental Sciences Research Center, Department of Endodontics, School of Dentistry, Guilan University of Medical Sciences, Rasht, Iran; 8 Faculty of Pharmacy and Pharmaceutical Sciences, Isfahan University of Medical Sciences, Isfahan, Iran; 9 Department of Physics, University of Zabol, P. O. Box. 98613-35856, Zabol, Iran

Retraction of: *Scientific Reports* https://doi.org/10.1038/s41598-022-13692-2, published online 08 June 2022

The Editors have retracted this Article.

After publication, concerns were raised the validity of the XRD spectra presented in Figure 1, and the authorship and author contributions. The Editors requested the authors to provide raw original data and explanations regarding the contributions, but found the response provided by the Authors insufficient. The Authors were also not able to provide all of the original data with meta-data that would allow for the verification of its veracity. Additionally, references 18–68 appear to be unrelated to the research described in this Article. The Editors therefore no longer have confidence in the reliability of the data presented in this Article.

Merhdad Khatami does not agree to this retraction. Maryam Darvish, Navid Nasrabadi, Farnoush Fotovat, Setareh Khosravi, Samira Jamali, Elnaz Mousavi, Siavash Iravani and Abbas Rahdar have not responded to any correspondence from the Editor about this retraction.

